# The Impact of Environmental and Material Factors on Fluoride Release from Metal-Modified Glass Ionomer Cements: A Systematic Review of In Vitro Studies

**DOI:** 10.3390/ma18133187

**Published:** 2025-07-05

**Authors:** Sylwia Klimas, Sylwia Kiryk, Jan Kiryk, Agnieszka Kotela, Julia Kensy, Mateusz Michalak, Zbigniew Rybak, Jacek Matys, Maciej Dobrzyński

**Affiliations:** 1 Department of Pediatric Dentistry and Preclinical Dentistry, Wroclaw Medical University, Krakowska 26, 50-425 Wroclaw, Poland; sylwia.klimas@student.umw.edu.pl (S.K.); s.roguzinska@gmail.com (S.K.); 2 Dental Surgery Department, Wroclaw Medical University, Krakowska 26, 50-425 Wroclaw, Poland; jan.kiryk@umw.edu.pl; 3 Medical Center of Innovation, Wroclaw Medical University, Krakowska 26, 50-425 Wroclaw, Poland; kotela.agnieszka@gmail.com (A.K.); mateusz.michalak92@gmail.com (M.M.); 4 Faculty of Dentistry, Wroclaw Medical University, Krakowska 26, 50-425 Wroclaw, Poland; julia.kensy@student.umw.edu.pl; 5 Pre-Clinical Research Centre, Wroclaw Medical University, Bujwida 44, 50-345 Wroclaw, Poland; zbigniew.rybak@umw.edu.pl

**Keywords:** fluoride release, glass-ionomer, metal-modified glass ionomer, silver, zinc, strontium, copper

## Abstract

Objective: Fluoride is widely recognized for its preventive role against secondary caries. This systematic review aimed to evaluate how environmental and material factors influence fluoride ion release from metal-reinforced glass ionomer cements. Methods: A structured literature search was performed in March 2025 across PubMed, Scopus, and Web of Science databases. Search terms included combinations of fluoride release AND glass ionomer AND silver OR zinc OR strontium OR copper. The study selection process followed PRISMA 2020 guidelines and was organized using the PICO framework. Out of 281 initially identified records, 153 were screened based on titles and abstracts. After applying predefined eligibility criteria, 23 studies met the inclusion requirements and were included in the qualitative analysis. Results: Among the 23 included publications, 12 involved glass ionomers modified with silver, and 6 of these reported an increase in fluoride release. Seven studies focused on zinc-modified cements, and four examined materials reinforced with strontium. Conclusions: The addition of strontium, titanium oxide, silver nanoparticles, or zirconium oxide increases the release of fluoride ions, while sintered silver reduces it. There is a great discrepancy among researchers regarding the effect of the addition of zinc oxide and its appropriate amount in the glass ionomer material.

## 1. Introduction

Fluoride release from dental materials plays a critical role in caries prevention and the long-term success of restorative treatments. Among various materials, metal-modified glass-ionomer cements (M-GICs) have attracted attention due to their ability to release fluoride over time, contributing to remineralization of adjacent tooth structures and inhibition of bacterial growth [1,2,3]. This ion-exchange mechanism helps protect the enamel and dentin, especially in patients at high caries risk. Fluoride ions can reduce demineralization by enhancing the formation of fluorapatite, which is more resistant to acid attacks [4,5,6]. Furthermore, fluoride possesses antibacterial properties, disrupting bacterial metabolism and reducing acid production [7,8]. However, the effectiveness of fluoride release depends on both material composition and environmental conditions, such as pH, temperature, and storage medium [9,10,11]. Recent studies have also explored the impact of surface coatings and nanomodifications on fluoride release profiles [12,13,14,15,16]. Variability in fluoride release among M-GICs has prompted interest in evaluating how different formulations and clinical factors influence this process [17,18]. Understanding these interactions is essential for optimizing material selection and enhancing therapeutic outcomes.

**Figure 1:**
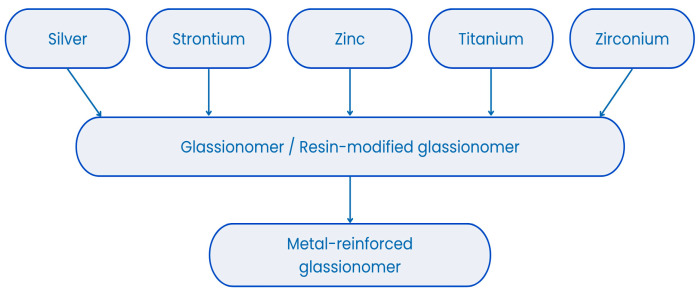
Metals present in metal-reinforced glass ionomers.

Metal-modified glass-ionomer cements (M-GICs) are hybrid dental materials developed to enhance the mechanical properties of glass-ionomer cements (GICs) [17,19]. They are composed of fluoroaluminosilicate glass powder and polyacrylic acid, with the addition of metal particles such as silver, titanium, or stainless steel (see Figure 1) to improve wear resistance, compressive strength, and radiopacity [20,21,22]. M-GICs maintain the essential features of traditional GICs, including chemical bonding to tooth structures, fluoride ion release, and biocompatibility, which contribute to caries prevention and enamel remineralization [20,23]. Their reinforced composition makes them suitable for use in stress-bearing areas, core build-ups, and atraumatic restorative treatment (ART) techniques, especially in pediatric and community dentistry [20,24,25]. The presence of metal may influence fluoride ion exchange and long-term release, which are key factors in their preventive function [12,26]. However, these materials may present esthetic limitations due to their opacity or metallic color, making them less ideal for anterior restorations [27]. Despite this, their durability, therapeutic ion release, and antimicrobial activity have made them valuable for clinical use [28]. Ongoing research explores nanomodifications and formulation adjustments to improve their physical, biological, and esthetic performance for broader indications [29,30,31,32,33,34,35].

The release of fluoride from dental materials used for tooth restoration plays a significant role in preventing and inhibiting the recurrence of caries [36,37,38]. The amount and dynamics of fluoride release are influenced by several factors. One of the most important of these is the pH of the environment in which the material is located. It has been proven that a decrease in pH increases the amount of fluoride ions released. This relationship is beneficial because increased activity of cariogenic bacteria triggers mechanisms that lead to the inhibition of their metabolism [39,40]. Another important factor is the medium in which the material is stored. Studies have shown that a greater amount of fluoride is released into deionized water than into artificial saliva. This is due to the influence of other ions on the solubility of dental materials [41,42]. Other important factors include the composition of the material and the concentration of the fluoride-containing substance. In the case of GIC-type materials, other factors include the porosity of the surface and the ability to charge fluoride, as well as the acid-base reaction at which a sudden release of large amounts of fluoride occurs due to its loose binding to cement [42,43]. In the case of resin-modified GICs and composite materials, fluoride release is influenced by the presence of the resin matrix, its hydrophilicity, the temperature at which the material is located, the size and amount of filler particles, and even the surface finishing technique of the filling [41,44,45,46].

While the release of fluoride ions from restorative dental materials is widely considered beneficial due to its cariostatic and antibacterial properties, it is equally important to take into account the potential risks associated with prolonged or excessive fluoride exposure. Although fluoride enhances remineralization and inhibits bacterial metabolism, excessive systemic intake may have adverse biological consequences. Elevated levels of fluoride have been shown to disrupt the mineralization of hard dental tissues, including both enamel and dentin, particularly during tooth development. This may result in dental fluorosis, which manifests as enamel hypomineralization and structural alterations in both enamel and dentin layers [47].

To ensure clinical safety, fluoride release from restorative materials should be carefully controlled and maintained within a therapeutic range that offers effective local action without contributing to systemic accumulation. Although glass ionomer cements typically exhibit an initial burst of fluoride release followed by a gradual decline, their long-term release patterns must be considered, especially in treatments involving young patients or those with multiple restorations [48]. These factors highlight the need for designing materials with predictable, sustained, and clinically appropriate fluoride release to balance caries prevention with overall biological safety.

This review aims to assess the influence of environmental and material factors on the release of fluoride from metal-modified glass ionomer cements. Having reviewed studies on this topic, we concluded that a comprehensive systematic review is needed. No such review was found in the literature. A detailed analysis of the factors responsible for the amount and mode of fluoride release is essential in order to understand the specificity of the type of glass ionomer cement being studied. This will help clinicians to select and use the material most efficiently in a clinical setting.

## 2. Materials and Methods

### 2.1. Focused Question

The systematic review followed the PICO framework [49] as follows. In the case of metal-modified glass ionomer cements (population), will exposure to various environmental and material factors (investigated condition) cause a change in fluoride release (outcome) compared to conditions without such influencing factors or compared to conventional glass ionomer cements (comparison condition)?

### 2.2. Protocol

**Figure 2:**
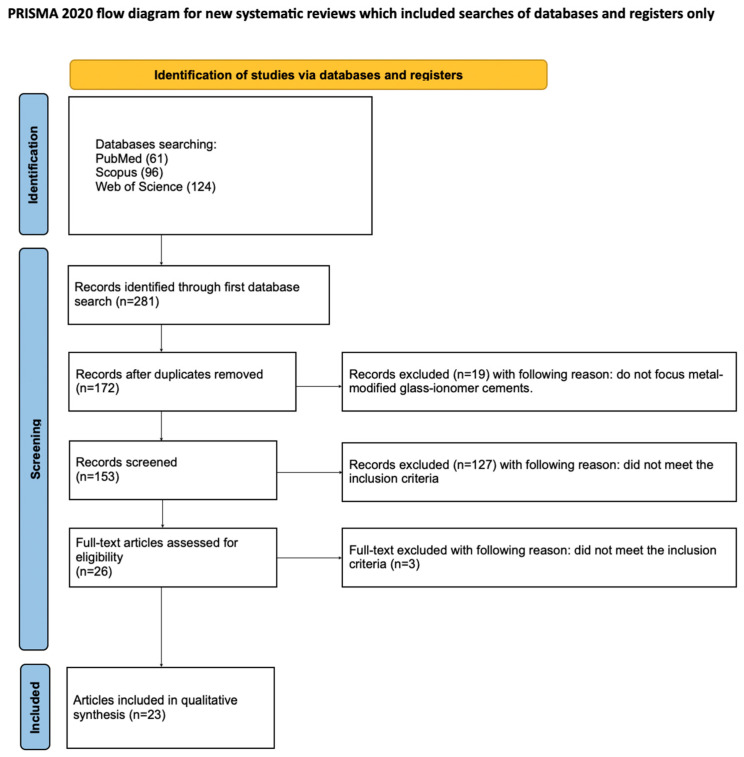
The PRISMA 2020 flow diagram.

The selection process for articles in the systematic review was carefully outlined following the PRISMA flow diagram [50] (see Figure 2). The systematic review was registered on the Open Science Framework under the following link: http://osf.io/7t5be (accessed on 26 May 2025). The completed checklist is provided in the Supplementary Materials (see Supplementary Table S1).

### 2.3. Eligibility Criteria

Studies were considered acceptable for inclusion in the review if they met the following criteria:

- Studies involving the examination of metal-modified glass-ionomer cements;
- Studies evaluating fluoride release;
- In vitro studies;
- Full-text articles
- Studies in English;

The exclusion criteria the reviewers agreed upon were as follows:

- Studies not focusing on glass-ionomers modified with metal/metal particles;
- Measurement of other properties than fluoride release;
- Non-English paper;
- Systematic review papers;
- Review articles;
- No full text accessible;
- Duplicated publications.

No restrictions were applied with regard to the year of publication.

### 2.4. Information Sources, Search Strategy, and Study Selection

Up to March 2025, an extensive literature search was performed using the PubMed, Scopus, and Web of Science (WoS) databases to identify studies that met the predefined inclusion criteria. The search was focused on exploring factors affecting fluoride content in tea infusions and was limited to titles and abstracts containing the keywords: (fluoride release AND glass ionomer) AND (silver OR zinc OR strontium OR copper). Articles were screened according to established eligibility criteria, and only studies with available full-text access were included in the final analysis.

### 2.5. Data Collection Process and Data Items

Six independent reviewers (S.K., J.K., A.K., J.K., S.K., and M.M.) independently screened and selected articles that fulfilled the inclusion criteria. Key data extracted from each study included the first author’s name, year of publication, study design, article title, metal type used to modify glass-ionomer cement, and measured fluoride content. All extracted information was systematically documented using a standardized Excel spreadsheet (Microsoft Excel 365, Version 2505, Build 16.0.18827.20102, 64-bit).

### 2.6. Risk of Bias and Quality Assessment

In the initial stage of study selection, all reviewers independently assessed titles and abstracts to minimize selection bias. Cohen’s kappa coefficient was used to evaluate the consistency of agreement between reviewers. Any discrepancies concerning the inclusion or exclusion of articles were resolved through group discussion among the authors [51].

### 2.7. Quality Assessment

The methodological quality of each included study was independently evaluated by two blinded reviewers (J.M. and M.D.) using the Joanna Briggs Institute (JBI) checklist for quasi-experimental studies (nonrandomized designs). The authors selected this tool because it fits the type of studies assessed, enables a reliable quality assessment, is methodologically recognized, enhances transparency and repeatability, and is consistent with international standards. This assessment tool consists of nine targeted questions, such as:

- Is it clear in the study what is the ‘cause’ and what is the ‘effect’?
- Were the participants included in any similar comparisons?
- Were the participants included in any comparisons receiving similar treatment/care, other than the exposure or intervention of interest? Was there a control group?
- Were there multiple measurements of the outcome both before and after the intervention/exposure?
- Was a follow-up completed, and if not, were differences between groups in terms of their follow-up adequately described and analyzed? Were the outcomes of participants included in any comparisons measured in the same way?
- Were the outcomes measured in a reliable way?
- Was an appropriate statistical analysis used?

Reviewers responded to each item using one of four options: “yes”, “no”, “unclear”, or “not applicable”. Any differing judgments were discussed until a mutual agreement was reached. To evaluate the consistency between raters, Cohen’s kappa was calculated using MedCalc (version 23.1.7, MedCalc Software Ltd., Ostend, Belgium). The resulting kappa coefficient was 0.84 (*p* < 0.001), signifying a very high level of agreement and reliability across reviewers.

## 3. Results

### 3.1. Study Selection

A total of 281 potentially relevant articles were identified through an initial search of PubMed, Scopus, and Web of Science. After duplicate removal, 172 records were retained for screening. An evaluation of titles and abstracts led to the exclusion of 146 articles that were unrelated to fluoride release from metal-reinforced glass ionomers. Of the 26 full-text articles assessed for eligibility, 3 were excluded for not meeting the inclusion criteria [33,52,53]. Consequently, 23 studies were included in the qualitative synthesis [54,55,56,57,58,59,60,61,62,63,64,65,66,67,68,69,70,71,72,73,74,75,76]. The high degree of variability among these studies precluded the possibility of conducting a meta-analysis.

### 3.2. General Characteristics of the Included Studies

Among the qualified works, the most frequently added and tested metal to glass ionomers was silver. Its compounds appeared in 12 publications [54,55,56,57,59,61,62,63,72,73,75,76]; in 7 studies, zinc compounds were used [59,61,64,65,66,67,74], and in 4, strontium [58,60,68,71]. Saxena et al. [70] studied the Zirconomer material in which zirconium oxide is present, and Cibim et al. [69] and Wassel et al. [62] experimentally added titanium oxide nanotubes to glass ionomer. Deionized water was standardly used as the sample storage environment [54,55,56,57,58,59,60,61,62,63,65,66,67,68,72,73,75,76]; four authors decided to use artificial saliva [64,70,74,76]; Shahid et al. [71] used acetic acid solution at pH 4; and Cibim et al. [69] used demineralizing and remineralizing solutions. The most frequently chosen measurement method was the use of an ion-selective electrode. It was used in 19 works [54,56,58,60,61,62,63,64,65,66,68,69,70,71,72,73,74,75,76]; in 2 studies, high performance liquid chromatography (HPLC) was used [57,59]; Guo et al. [55] used an ion chromatograph (IC); and Putri et al. [67] used a spectrophotometer. Some researchers decided to measure the release of other ions, apart from fluorine, and so in four works the level of zinc [64,65,66,74] was checked; in six, calcium [57,58,60,66,68,71]; in five, aluminum [57,58,60,66,71]; in two, silicon [60,66]; in three, strontium [58,66,71], in two, sodium [57,66], in three, phosphorus [57,58,66], and in two, silver [54,57]. Pardi et al. [54] additionally studied the release of vanadium, and Bahammam et al. [66] the release of oxygen, magnesium, sulfur, and zirconium (see Table 1).

### 3.3. Main Study Outcomes

#### 3.3.1. Influence of Silver Additions on Fluoride Release

Four publications investigated the Ketac Silver material from GC, which contains sintered silver [72,73,75,76]. Three of these reported lower fluoride release compared to glass ionomers without silver [72,75,76]. Hattab et al. [76] even observed a fourfold decrease. In contrast, Xu et al. [73] reported higher fluoride release, although accompanied by poorer mechanical properties. In studies where silver nanoparticles were used, five authors reported an increase in fluoride release [54,56,59,61,62]. However, in AlMatar et al. [59], this effect was observed in combination with ZnO, and Wassel et al. [62] combined silver with TiO_2_. Pardi et al. [54] and Raghimi et al. [56] found increased fluoride only at specific concentrations. Conversely, Guo et al. [55], Alshehri et al. [63], and Qasim et al. [57] did not observe improvement or reported reduced fluoride release.

#### 3.3.2. Influence of Zinc Oxide and Its Compounds

Studies by Gunay et al. [61] and Bahammam et al. [66] showed that zinc addition decreases fluoride release. Malekhoseini et al. [65] confirmed this at 3% ZnO, while 2% led to increased release. Putri et al. [67] observed an increase at 10% ZnO, with no further effect at higher levels. Osinaga et al. [74] found no effect of ZnSO_4_ on fluoride release, though antibacterial properties improved. Kohno et al. [64] concluded that the fluoride level was insufficient for bacterial inhibition.

#### 3.3.3. Influence of Strontium Additions

All studies involving strontium-modified glass ionomers consistently reported increased fluoride release [58,60,68,71]. For example, Karimi et al. [68] observed higher fluoride levels with increased concentrations of strontium-containing ACP nanoparticles. Shahid et al. [71] measured fluoride release across various combinations of SrO and SrF_2_ and found a clear trend: groups with 1.5% SrF_2_ and 1% SrO showed the highest cumulative fluoride values (2.61 mequiv/g). Thongsri et al. [60] also demonstrated that materials with 1% bioactive strontium glass had improved fluoride rerelease and better compressive strength than the control group. Overall, strontium-modified GICs showed consistent improvements in fluoride release across multiple studies and conditions.

#### 3.3.4. Influence of Titanium Dioxide

Cibim et al. [69] examined the effect of TiO_2_ nanotube additions (3%, 5%, and 7%) in both demineralizing and remineralizing solutions. They found that fluoride release increased at 3% and 7% concentrations, but not at 5%. In demineralizing solution, the 3% group reached 1.172 ppm on day 15, the highest recorded in their experiment. Surface hardness also improved in the 5% group (118.25 ± 4.21 HV). Wassel et al. [62] tested a GIC modified with 5% TiO_2_ and reported slightly reduced cumulative fluoride release (0.047 mg/cm^2^) compared to the AgNP-modified version (0.065 mg/cm^2^), but compressive strength was significantly higher (166.31 ± 15.08 MPa). These results suggest that TiO_2_ may enhance mechanical properties while having a variable effect on fluoride release depending on concentration and experimental conditions.

#### 3.3.5. Influence of Zirconium Dioxide

Only one study by Saxena et al. [70] investigated GIC modified with zirconium oxide (Zirconomer). The fluoride release was significantly higher at all time points compared to Fuji IX. For instance, on day 7, Zirconomer released 35.65 ppm versus 15.46 ppm from Fuji IX. The material also showed greater antibacterial activity against *Streptococcus mutans* and *Lactobacillus casei*. However, no data on mechanical properties were provided. Given that this is a single study, the promising results should be interpreted cautiously until validated by further research.

#### 3.3.6. Comparative Summary

The studies suggest that the addition of metals may either enhance or reduce fluoride release, depending on their type, form, and concentration. The most consistent positive effect was seen with strontium, which reliably increased fluoride release across all studies. Titanium dioxide also showed promise, particularly when used at specific concentrations. Zirconium oxide demonstrated the highest fluoride release among compared materials in a single study. In contrast, the effects of silver and zinc varied significantly depending on their chemical form and concentration. These results highlight the importance of selecting additives based on both the desired fluoride release profile and the mechanical performance of the final material. The findings summarized here are supported by a wide range of studies covering various metal modifications and testing conditions, including those by Pardi et al. [54], Guo et al. [55], Raghimi et al. [56], Qasim et al. [57], Potiprapanpong et al. [58], AlMatar et al. [59], Thongsri et al. [60], Gunay et al. [61], Wassel et al. [62], Alshehri et al. [63], Kohno et al. [64], Malekhoseini et al. [65], Bahammam et al. [66], Putri et al. [67], Karimi et al. [68], Cibim et al. [69], Saxena et al. [70], Shahid et al. [71], Selimovic-Dragas et al. [72], Xu et al. [73], Osinaga et al. [74], Helvatjoglu-Antoniades et al. [75], and Hattab et al. [76] (see Table 2).

### 3.4. Quality Assessment

For all of the 9 questions, there were 2 papers that scored maximum points and received a positive answer to 9 of them [54,70] and the remaining 21 papers received a positive answer to 8 of them [55,56,57,58,59,60,61,62,63,64,65,66,67,68,69,71,72,73,74,75,76] (see Table 3).

## 4. Discussion

This study aims to assess how both the material composition and external environmental conditions influence the fluoride release behavior of metal-reinforced glass ionomer cements (MGICs). Our synthesis of 23 studies demonstrated that the impact of metal incorporation is highly dependent on the type, form, and concentration of additives [54,55,56,57,58,59,60,61,62,63,64,65,66,67,68,69,70,71,72,73,74,75,76]. The data suggest that while certain modifications, such as optimal levels of silver or titanium nanoparticles, may promote fluoride release [54,56,69], others, like sintered silver or excessive strontium content, may reduce it [68,72,75,76]. Both Pardi et al. [54] and Hattab et al. [76] reported early peaks in fluoride release, with Pardi observing a maximum on day 7 and Hattab noting high initial values that stabilized after two weeks. This behavior suggests that short-term efficacy may be overestimated if cumulative release profiles are not taken into account. The synthesis highlights dose- and formulation-dependent patterns across diverse experimental settings, such as distilled water, artificial saliva, or acidic environments [54,55,56,57,58,59,60,61,62,63,64,65,66,67,68,69,70,71,72,73,74,75,76]. Differences in ion release behavior observed under deionized water versus artificial saliva further illustrate the complexity of translating lab data to clinical conditions [76]. Overall, the findings underscore that fluoride release cannot be attributed to metal addition alone, but to its integration into specific glass matrices, setting mechanisms, and environmental exposures.

Our analysis showed that cermet cements containing sintered silver consistently exhibit reduced fluoride release compared to other glass ionomers. In three studies, Ketac Silver demonstrated notably lower cumulative fluoride values [72,75,76]. Hattab et al. [76] reported a fourfold decrease in release versus Ketac-Fil and Fuji II in deionized water, while Helvatjoglu-Antoniades et al. [75] observed only 3.1 µg/mm^2^ from Ketac Silver compared to 8.3 and 11.7 µg/mm^2^ in Fuji III and Miracle Mix, respectively. Xu et al. [73] found a relatively higher release (318 µg/cm^2^—cumulative fluoride release in 21 days), yet still below Miracle Mix and coupled with reduced compressive strength. These results, consistent across durations and media, suggest that cermets may underperform in fluoride-based prevention. Similarly, Yip et al. [77] also reported that the cermet Ketac Silver released less fluoride than other glass ionomer cements, a finding supported by Wandera et al. [78], who observed lower fluoride release from Ketac Silver compared to Ketac-Fil in both distilled water and artificial saliva. Originally developed for improved strength, sintered silver cermets may offer limited caries-inhibitory benefits and should be reconsidered for high-risk patients due to low fluoride release and poor recharge capacity.

The current synthesis establishes that additive effects on fluoride release are not uniform and depend on chemical formulation and dosing. Silver nanoparticles appear to enhance fluoride release primarily within specific concentration ranges, as shown in materials modified with AgVO_3_ or Ag/HA/Si [54,56]. In Pardi et al.’s study [54], fluoride release peaked at day 7 in the 1% and 2.5% groups, then declined. In contrast, Raghimi et al. [56] demonstrated statistically significant improvements at 1% and 2%, with plateauing at higher doses. However, combinations involving zinc oxide or titanium dioxide did not always result in further increases [59,62]; in fact, AlMatar et al. [59] found that the simultaneous addition of 5% ZnONP and 5% AgNP reversed the fluoride gain achieved by 5% AgNP alone. According to the findings of Wasel et al. [62], although the addition of 5% Ag-NP did not lead to a statistically significant increase in fluoride release compared to the control group, a significantly higher fluoride release was observed in comparison with the TiO_2_-NP group. Zinc showed a threshold-dependent trend: 2% ZnO increased fluoride release, whereas 3% caused a marked decline [65]. Putri et al. [67] reported similar results with ZnO at 10% enhancing, but 15% reducing fluoride release. Titanium dioxide was effective at 3% and 7% but not 5% [69]. Strontium compounds varied: while Shahid et al. [71] reported a large increase at specific SrF_2_/SrO ratios (1.5%/1%), Karimi et al. [68] found a decrease in fluoride with increasing Sr content. The study by Saxena et al. [70] showed that GIC containing ZrO_2_ (Zirconomer) released higher amounts of fluoride compared to the conventional GIC (Fuji IX) at all time points. This indicates that the incorporation of ZrO_2_ enhances fluoride ion release and prolongs its availability over time.

Notably, the findings contextualize fluoride release in relation to potential antibacterial and remineralizing functions. Based on the study by Malekhoseini et al. [65], the addition of 2% ZnO to RMGI cement provided the strongest antibacterial effect and the highest fluoride release. Increasing the concentration to 3% did not enhance antibacterial activity and reduced fluoride release, indicating a threshold beyond which performance may decline. Guo et al. [55] demonstrated reduced biofilm formation with AgNW-modified GICs despite unchanged fluoride levels. Osinaga et al. [74] reported that ZnSO_4_-modified GICs showed improved antibacterial properties without observable negative effects on overall fluoride release patterns. Zirconomer exhibits stronger antibacterial effects against *Streptococcus mutans* and *Lactobacillus casei*, along with a higher level of fluoride release [70]. According to Cibim et al. [69], incorporating 5% TiO_2_ into GIC enhanced its fluoride ion release capacity. Alshehri et al. [63] further showed that silver-modified GICs did not enhance fluoride recharging or remineralization potential. Kohno et al. [64] confirmed that fluoride concentrations in Zn-containing GICs were insufficient to inhibit biofilm growth. According to the study by Wassel et al. [62], glass ionomers modified with Ag and TiO_2_ exhibited greater antibacterial activity, as evidenced by larger inhibition zones compared to conventional GICs. In the study by Gunay et al. [61], Riva Silver and ChemFil Rock showed markedly stronger antibacterial activity than the conventional glass ionomer Ketac Molar Easymix. Importantly, the evidence underscores that while fluoride plays a significant role, it cannot serve as the sole predictor of clinical benefit. Therefore, interpreting biological effects should include considerations of ion release synergy, local pH, and material structure.

Numerous studies have confirmed that the incorporation of nanoparticles into glass ionomer cements (GICs) can significantly alter their mechanical properties, often in a trade-off-dependent manner. Guo et al. [55] observed that silver nanowires and nanoparticles decreased both compressive strength and microhardness at all tested concentrations. AlMatar et al. [59] reported that combining AgNP and ZnONP enhanced fluoride release but lowered microhardness compared to the control. Cibim et al. [69] found that TiO_2_ at 3–5% increased surface hardness, but 7% reduced it; surface roughness showed an increasing trend with higher TiO_2_ concentrations, though the relationship was not linear. In the study by Raghimi et al. [56], compressive strength improved up to 1% Ag/HA/Si but dropped at 2%. Similarly, Karimi et al. [68] reported that increasing Sr content enhanced compressive strength up to a point, but this coincided with reduced fluoride release at higher doses. Wassel et al. [62] showed that TiO_2_NP addition increased compressive strength without drastically changing fluoride output. Meanwhile, Potiprapanpong et al. [58] demonstrated that Sr/F-BGNPs improved fluoride release, but the formulation with the highest release (H5S10) had the lowest biaxial flexural strength. Finally, Osinaga et al. [74] showed slight decreases in flexural strength with ZnSO_4_-modified GICs. Collectively, these findings highlight the need to balance mechanical enhancement with functional efficacy when designing nanoparticle-modified GICs.

Based on the reviewed studies, several modified glass ionomer cements appear promising for future clinical use due to their enhanced fluoride release and favorable supplementary properties. RMGICs modified with strontium/fluoride-containing bioactive glass nanoparticles and HEMA demonstrated high fluoride release and good biocompatibility [58]. Although the formulation with 10% Sr/F-BGNPs released slightly more fluoride, the 5% Sr/F-BGNPs variant offered a better balance between ion release and mechanical strength. Given its comparable fluoride performance and superior flexural properties, the latter may be more appropriate for patients with parafunctional habits, high occlusal loads, or advanced tooth wear. Similarly, the addition of Ag/HA/Si hybrid nanoparticles [56] or 2% ZnO nanoparticles [65] to RMGICs significantly increased fluoride ion release; however, the 2% Ag/HA/Si concentration slightly reduced compressive strength compared to the unmodified control, suggesting that lower concentrations—1% may offer a better balance between mechanical integrity and fluoride release. GICs modified with 5% TiO_2_ nanoparticles showed enhanced surface hardness and fluoride release [69], and Zirconomer displayed strong antibacterial effects along with sustained fluoride release [70]. Additionally, GICs containing silver nanowires effectively reduced biofilm adherence while maintaining stable fluoride profiles [55]. These findings suggest that such formulations may offer improved therapeutic potential, particularly in high-caries-risk patients, individuals with poor oral hygiene, or pediatric patients. Their properties also make them a promising option for managing cases with multiple restorations or a history of recurrent caries.

This systematic review included studies conducted in vitro, which do not fully reflect the complex dynamics of the oral environment, including factors such as temperature fluctuations, enzymes, bacterial biofilm, and variable pH. The effect of fluoride in the oral cavity is more distributed over time and is subject to fluctuations due to the medium of saliva containing proteins and ions. Its composition and quantity change over time, which affects the solubility and diffusion of fluoride. In addition, in vitro studies of dental materials neglect long-term factors such as microcracks, abrasion, bacterial infiltration, and thermal cycling. The heterogeneity of experimental protocols, including variations in storage media, measurement intervals, and fluoride detection methods, complicates direct comparisons between studies. Moreover, many articles assessed only short-term fluoride release (up to 15 days) [55,59,60,64,65,66,67,69,71,72], which may not accurately represent long-term clinical performance. The influence of biofilm presence on fluoride release remains insufficiently explored. Therefore, it is imperative that future studies include in situ models or are conducted in clinical settings, taking into account bacterial biofilm, pH, and storage media to better mimic oral conditions. Additionally, employing multi-species biofilm models instead of single-strain bacterial cultures could improve the understanding of antimicrobial behavior in more realistic conditions. Furthermore, it is recommended to use long-term observations and standardized measurement methods. Investigating the relationship between fluoride release, mechanical durability, and antibacterial activity over time would be particularly valuable for the development of optimized metal-modified glass ionomer cements (MGIC). Such approaches would help reduce discrepancies between in vitro results and actual clinical outcomes, ultimately supporting the development of more effective restorative materials.

## 5. Conclusions

The analysis of the studies showed that adding silver in the form of nanoparticles in the amount of 2–2.5% by weight to the glass ionomer increases fluoride secretion, but sintered silver has the opposite effect. Even more fluoride can be obtained by adding titanium oxide together with silver nanoparticles, but this may have a negative effect on the mechanical parameters of the material. In turn, the addition of zinc oxide, in addition to increasing fluoride secretion, may bring additional benefits in the form of antibacterial action, but further research is necessary in this area due to large discrepancies between studies regarding the appropriate proportions, and it has been proven that the incorrect amount of zinc oxide can have exactly the opposite effect. The most reliable additives in terms of positive effects in clinical practice seem to be strontium, titanium oxide, and zirconium oxide. The effect of strontium is confirmed by numerous studies, but further analyses of the effect of titanium oxide and zirconium oxide are necessary due to the insufficient number of publications in this area.

## Figures and Tables

**Table 1:** General characteristics of studies.

Study	Aim of the Study	Material and Methods	Results	Conclusions
Pardi [54]	Assessment of the surface characteristics and ion release behavior of glass ionomer cement (GIC) modified with nanostructured silver vanadate (AgVO_3_).	GIC samples prepared with AgVO_3_ concentrations of 0% (control), 1%, 2.5%, and 5%. Fluoride release measured via ion-selective electrode; surface distribution assessed by SEM/EDS; silver and vanadium ion release quantified using ICP-MS.	Fluoride release peaked at day 7 in all groups and then declined by day 28. 1% and 2.5% AgVO_3_-modified samples showed higher fluoride release than unmodified samples. Silver release increased notably in the 2.5% and 5% groups. The highest vanadium release occurred at 5% concentration.	The introduction of AgVO_3_ altered both the surface characteristics and the ion release profile of glass ionomer cement (GIC). The addition of AgVO_3_ at concentrations of 1% and 2.5% initially promoted an increase in fluoride release.
Guo [55]	Assessing antibiofilm properties and mechanical/biochemical performance of glass ionomer cement modified with silver nanowires (AgNWs).	6 GIC groups were tested: control (no nanosilver), four AgNW concentrations (0.05–0.5 wt%), and 0.5 wt% AgNP (positive control). Analyses included fluoride release (IC: ICS-6000), biofilm formation, lactic acid production, mechanical properties, color stability, and cytotoxicity.	Fluoride release and lactic acid levels showed no significant differences across samples. AgNW-containing GIC reduced biofilm adherence versus conventional GIC.	Fluoride ion release remained stable across all GIC materials, with no significant differences between groups or time intervals.
Raghimi [56]	Assessment of fluoride release and mechanical performance of glass ionomer cement containing hybrid nanoparticles composed of silver hydroxyapatite and silica.	60 cylindrical samples were prepared using BracePaste composite, pure RMGI (GC Fuji II LC), and RMGI with Ag/HA/Si hybrid nanoparticles (0.1–2 wt%). Fluoride release was measured via an ion-selective electrode, and compressive strength (MPa) via a universal testing machine.	Resin-modified glass ionomer containing 1% (0.21 ± 0.07 mg/mL) and 2% (0.45 ± 0.22 mg/mL) hybrid nanoparticles exhibited a significantly higher fluoride release compared to the control group (0.09 ± 0.03 mg/mL). Ag/HA/Si hybrid nanoparticle addition did not significantly affect compressive strength.	Fluoride release increased with higher Ag/HA/Si hybrid nanoparticle concentrations in RMGIC. This enhancement suggests improved potential for dental applications.
Qasim [57]	To assess how mesoporous silica with silver nanoparticles affects properties and ion release in conventional glass ionomer cements.	Conventional GICs were modified with mesoporous silica containing silver nanoparticles (1–5%). Fluoride release was measured via HPLC (Prominence Shimadzu), and other ions (Al, Ca, Na, P, Ag) via ICP-OES. Additional tests included NanoCT, FTIR, surface microhardness, water sorption, and solubility.	Control specimens showed the highest fluoride release, followed by the 5%, 3%, and 1% groups. Significant differences existed between control and 5% specimens during weeks 1–4, with no significant differences within groups. Modified GICs demonstrated similar microhardness values to conventional ones.	Mesoporous silica with silver nanoparticles reduced fluoride release in glass ionomer cement compared to conventional material, though release increased with higher additive content.
Potiprapanpong [58]	To analyze the mechanical behavior, chemical composition, and biocompatibility of RMGICs enhanced with strontium/fluoride bioactive glass nanoparticles and polyacids functionalized with methacrylate groups.	RMGICs were mixed with HEMA and spherical Sr/F-bioactive glass nanoparticles. Fluoride release was measured over 4 weeks via ion-selective electrode (Orion) in deionized water (37 °C). Al, Ca, P, and Sr concentrations were assessed after 4 weeks using ICP-OES (Optima 8300). Setting reaction, BFS, BFM, and cytotoxicity were also evaluated.	All RMGICs showed time-dependent increases in cumulative fluoride release. Materials with 5% HEMA and either 10% or 5% Sr/F-BGNPs had similar highest release values (137.5 ± 6.1 ppm and 136.6 ± 2.2 ppm, respectively). HEMA addition increased average cumulative fluoride release by 89.11%, while higher Sr/F-BGNPs concentrations had minimal effect.	HEMA addition and higher concentrations (10%) of spherical Sr/F-bioactive glass nanoparticles enhanced cumulative fluoride release. Modified RMGIC formulations released more fluoride than commercial material.
AlMatar [59]	Evaluation of the effect of adding SNP (silver) and ZnONP (zinc oxide) to the RMGIC structure on fluorine release.	The samples were prepared by adding to RMGIC 1. 5 wt% SNP 2. 5 wt% ZnONP 3. 5 wt% SNP and ZnONP (1:1). The samples were cured and examined: under SEM, spectroscope (FTIR), nanotomography, Vickers hardness test, and HPLC.	ZnONP and ZnONP+SNP increased sample roughness (SEM/nanotomography). SNP and ZnONP+SNP decreased hardness. SNP and SNP+ZnONP samples showed increased F release at specific intervals versus control.	Adding silver or zinc nanoparticles to RMGIC in combination with SNP increases the release of F from the material.
Thongsri [60]	To investigate whether the addition of strontium-containing bioactive glass (BGF) to sol-gel GI affects the absorption and release of fluoride from the material.	Bioactive glass containing strontium (BGF) was added to sol-gel GI (SCGI) in amounts of 0, 1, 3, and 6 wt%. The release and absorption of F in the samples was assessed using: FISE, SEM-EDS, and XPS. Cytotoxicity, setting time, and resistance to compression were also tested.	BGF >1% increases setting time and reduces compressive strength; only BGF 1% improves CS. SCGI and SCGI+BGF1% show higher F uptake/rerelease than commercial GI, which has greater initial F release. SCGI+BGF toxicity is comparable to commercial GI.	The addition of bioactive glass containing strontium in an appropriate amount to GI can improve its F-releasing and absorption properties and biomechanical properties without affecting toxicity.
Gunay [61]	Evaluation of four different glass ionomers regarding their F release, antibacterial properties, and cytotoxicity.	200 samples of 4 glass-ionomers (Riva Silver with silver, Equia Forte HT glass hybrid, ChemFil Rock with zinc, and KetacTM Molar Easymix) were tested for: antibacterial properties (bacterial growth assessment), cytotoxicity (WST-1 analysis with mouse fibroblasts), and F release (ion-selective electrode, Thermo Orion 720 A+, measured on days 1, 2, 3, 7, 14, 21, 28).	Ketac TM Molar released the most fluoride. ChemFil Rock released the least fluoride. GI with silver released more fluoride than GI with zinc, but less than classic GI-Ketac.	The best GI in terms of fluoride release is GI without added metal ions and glass.
Wassel [62]	Evaluation of how the addition of Ti Ag ions to GI will affect its antibacterial, mechanical, and fluoride-releasing properties.	10 samples were prepared per parameter for conventional GI (control), GI + 5%wt Ag-NP, and GI + 5%wt TiO_2_-NP. Tests included: antibacterial properties (inhibition zones), fluoride release (selective electrode Orion Research, Inc., measured at 24 h, 14 and 28 days), and compressive strength (load at fracture, MPa).	GI with Ag and TiO_2_ produced larger growth inhibition zones than conventional GI samples. GI with Ag released the most F, GI with TiO_2_ the least over 28 days. CS values were significantly higher for TiO_2_ samples than for GI with Ag and conventional GI.	The inclusion of Ag and Ti ions in the GI structure improves the tested material parameters.
Alshehri [63]	Evaluation of the effect of adding silver to GI on fluoride ion release and recharging parameters.	60 samples in 6 groups were tested: conventional GIC, GIC-Ag 0.1%, GIC-Ag 0.2%, conventional GIC + F, GIC-Ag 0.1% + F, and GIC-Ag 0.2% + F. Groups 4-6 received fluoride loading from 1450 ppm paste. Fluoride uptake and recharging were measured using FISE (HI4110, Hanna Instruments) on days 1, 2, 7, 15, and 30.	Conventional GICs released the most fluoride in both non-loaded (group 1) and F-loaded (group 4) categories. Non-loaded GICs released more fluoride than their F-loaded counterparts.	Adding Ag ions to GI did not improve the remineralizing properties of the material.
Kohno [64]	To investigate the possibility of loading and releasing Zn^2+^ and F^−^ from GIC containing BioUnion filler.	Artificial saliva was dripped onto the GIC containing BioUnion filler and periodically replaced with acetic acid. The release/loading of Zn^2+^ and F^−^ ions was checked by measuring their concentrations.	The concentration of released Zn^2+^ and F^−^ was higher in acid than in artificial saliva. However, in none of the GICs was the concentration of released F^-^ sufficient to inhibit bacterial biofilm.	GIC containing BioUnion filler releases Zn^2+^ and F^−^ ions and can also be recharged by applying a tooth gel containing Zn^2+^ and F^−^.
Malekhoseini [65]	Evaluation of the effect of different concentrations of ZnO nanoparticles in RMGI on mechanical and antibacterial properties.	100 glass ionomer samples with ZnO concentrations of 0%, 1%, 2%, 3%, or 4% were prepared. The concentration of Zn and F ions released from them was measured using a spectrophotometer or IPC, respectively.	The lowest level of fluoride was released from the glass ionomer containing 3% ZnO, and the highest from the RMGIC containing 2% ZnO.	Adding 2% ZnO nanoparticles to RMGI significantly increases fluoride release and antibacterial activity without affecting mechanical parameters.
Bahammam [66]	To investigate the fluoride release over a 9-day period from four glass ionomer cements.	Disks were made of ChemFil ROCK, Fuji IX, Riva self-cure, and Ketac Nano materials. They were immersed in distilled water, and fluoride release was measured using energy dispersive spectrometry.	The first-day fluoride release of Fuji IX was significantly higher than that of Ketac Nano, Riva self-cure, and ChemFil ROCK.	Among glass ionomer restorative cements, there is a wide range of fluoride ion release, with the highest level of release on the first day observed with Fuji IX cement, followed by Ketac Nano, Riva self-cure, and ChemFil ROCK.
Putri [67]	To investigate the effect of ZnO addition to GIC on the release of fluoride ions.	In group I, 1 g of ZnO was added to 9 g of glass ionomer powder, and in group II, 1.5 g of ZnO was added to 8.5 g of powder. Samples were prepared by mixing them with glass ionomer liquid. They were immersed in distilled water for 24 h, and then the level of released fluorine was tested.	Adding 1 g of zinc oxide nanoparticles can significantly increase the fluoride release of GIC. Meanwhile, adding 2 g of zinc oxide nanoparticles cannot increase the fluoride release of GIC.	Adding 1g of ZnO to the glass ionomer increases the fluoride release.
Karimi [68]	To find the optimal dose of ACP to add to RMGIC to activate alkaline phosphatase (ALP) and osteogenic differentiation of human mesenchymal stem cells (hMSC) without compromising GIC properties.	A GIC powder consisting of melt-derived strontium fluoro-aluminosilicate glass (SFAG) and synthetic ACP nanoparticles was created. This was combined with a commercial glass ionomer liquid, and a sample was made. The amount of fluoride released was then checked after 28 days of soaking in distilled water.	Addition of ACP to GIC up to 5.0% does not significantly reduce the release of F^−^.	ACP nanoparticles improved ALP activity and hMSC differentiation at the cost of negligible changes in fluoride release rate and compressive strength.
Cibim [69]	Evaluation of the effect of TiO2 addition to GIC on its physicochemical and biological properties.	Samples were prepared from GIC powder with TiO2 added in different proportions mixed with GIC liquid. They were stored in a demineralizing and remineralizing solution, changing it every 24 h. The amount of released fluoride was checked after 15 days.	All TiO2 groups released more fluoride compared to the control group, except KM + 3% TiO2.	The addition of 5% TiO2 to GIC increased the non-collagenous composition of the ECM improved microhardness and fluoride release capacity without affecting the surface roughness.
Saxena [70]	Comparison of powder composition, fluoride release, and antimicrobial properties of atraumatic zirconia restorative material with conventional GIC.	Zirconomer and Fuji IX samples were prepared and stored in artificial saliva. Fluoride content in the sample was tested after 24 h, 3, 7, 15, and 30 days.	In each of the measurements, Zirconomer showed significantly higher amounts of released fluoride.	Zirconomer has greater antibacterial activity against *Streptococcus mutans* and *Lactobacillus casei*, and releases more fluoride. However, it does not have antifungal activity against *Candida albicans.*
Shahid [71]	To evaluate the effect of replacing CaO and CaF2 with SrO and SrF2 in glass ionomer powder on the aesthetics and ion release of the finished GIC.	Samples were prepared from ionic glasses with different contents of Sr, Ca, and F. They were stored in acetic acid at pH 4.0 for 7 days at 37 °C. Then the contents of F^−^, Sr^2+^, Ca^2+^, and Al^3+^ ions in the solutions were examined.	The release of fluoride ions and all cations is linear with time, indicating diffusion control. This is consistent with the pH of the acidic medium and its changes.	Replacing calcium with strontium increases fluoride release by GIC.
Selimovic-Dragas [72]	To determine the amount of fluoride released from GIC and RMGIC and its influence on the cytotoxicity of these materials.	Samples were prepared from GC Fuji IX GP Fast, GC FUJI Triage, Ketac Silver, GC Fuji II LC, GC Fuji Plus, and Vitrebond and stored in distilled water. Fluoride measurements were taken after 8 and 24 h.	After both 8 and 24 h, Ketac Silver released the least fluoride of all materials tested.	Silver-doped GIC released less fluoride than conventional and resin-modified glass ionomer materials.
Xu [73]	To assess compressive strength, fluoride release, and recharge of 15 dental materials and explore correlations between these properties.	15 fluoride-releasing materials (various glass ionomers, compomers, cermets, and composites) were tested. Cylindrical specimens were light-cured per manufacturer instructions. Compressive strength was measured at 24 h (Instron). Fluoride release was monitored daily for 21 days, with rechargeability tested after 3 months using 2% NaF foam.	Miracle Mix had the highest fluoride release (398 mg/cm^2^/21 days), exceeding Ketac-Silver (318 mg/cm^2^) especially in the first 4 days, but both showed lower strength than conventional GICs. Resin-modified GIs (led by Photac-Fil) had better strength than conventional GICs. Compomers/composites showed the highest strength and lowest fluoride release, except for Solitaire (422 mg/cm^2^).	Materials combining strong fluoride release with good mechanical properties remain limited. Resin-modified glass ionomers provide the best balance for high-caries-risk patients.
Osinaga [74]	Evaluating how the addition of zinc sulfate (ZnSO_4_) to GIC and RMGIC affects its physical, antibacterial, and F and Zn release properties.	Ketac-Fil (conventional GIC) and Vitremer (RMGIC) were modified with 0%, 5%, or 10% ZnSO_4_. 72 samples stored in artificial saliva were tested for F and Zn release over 30 days (days 16–30 with recharging) using ion-selective electrodes. Solubility, flexural strength, and antibacterial properties were also evaluated.	F release peaked on day 1, then decreased and stabilized, with a temporary spike after day 15 recharging. 10% ZnSO_4_ samples released most Zn (0.9 ± 0.5 and 7.5 ± 0.4 ppm) and were higher in Vitremer than Ketac-Fil. Zn release was highest in the first 24 h, minimal thereafter, with no post-recharge increase. Higher ZnSO_4_ increased solubility without affecting flexural strength. 10% ZnSO_4_ showed best antibacterial effect.	The addition of ZnSO_4_ to GICs improved antibacterial properties against *S. mutans* without negatively impacting their physical characteristics or fluoride release patterns.
Helvatjoglu-Antoniades [75]	Assessment of the amount of fluoride released from different restorative materials.	9 materials were tested: four GICs (Miracle-Mix, Fuji Type III, Fuji II LC, and Ketac-Silver), Ketac-Cem luting cement, Compoglass Flow compomer, two sealants (Fissurit F, Helioseal F), and Tetric composite. Seven samples per material were placed in 7mL double-distilled water at 37 °C. Fluoride release was measured via ion-selective electrode at 4 h to 112 days.	All materials showed high initial fluoride release (first 24 h), followed by substantial decrease and gradual prolonged release. Glass ionomers released more fluoride than composites. Release ranking: Miracle Mix > Fuji III/Ketac Cem > Fuji II LC > Ketac Silver/Compoglass Flow > Fissurit F/Helioseal F > Tetric. 50% of cumulative release occurred in the first week.	Fluoride release was observed in all materials over the entire 16-week period. Glass ionomers and compomers exhibited higher fluoride release levels compared to sealants and composite resins.
Hattab [76]	To investigate the fluoride release from GIC, comparing release in deionized water and artificial saliva.	54 samples each of Ketac-Fil, Fuji II, and Ketac-Silver were divided into uncoated, varnish-coated, and resin-coated groups. Each was tested in deionized water, artificial saliva (pH 5.5), or hydroxyapatite suspension (50 mL, 37 °C). Fluoride release was measured via specific electrode over 28 days at intervals from 1 h to weekly.	Conventional GICs released 4x more fluoride than Ketac-Silver. All showed high initial release, stabilizing after 2 weeks. Artificial saliva reduced release versus water; coatings decreased release 27.5–79.9%. Hydroxyapatite absorbed nearly all fluoride. Only small percentages (1.0–3.8%) of total fluoride were released over 28 days.	GIC released less fluoride in artificial saliva than in deionized water, and surface coatings reduced its fluoride release.

**Table 2:** Detailed characteristics of included studies.

Author	Type of GIC Cement	Type of Additive/Metal	Amount/Concentration of Additive (%)	Fluoride Measurement Method	Storage Environment	Total Fluoride (ppm)	Total Fluoride (ppm)	Mechanical Parameters—Numerical Values
Pardi [54]	Riva Self Cure (conventional GIC)	AgVO_3_-nanostructured silver vanadate	0, 1, 2.5 and 5 wt%	FISE (Fluoride Ion-Selective Electrode)	deionized water	Riva Self Cure 0% Day 1: 10 ± 1 ppm Day 7: 15 ± 1 ppm Day 14: 10 ± 3 ppm Day 21: 5.9 ± 0.7 ppm Day 28: 4 ± 1 ppm Riva Self Cure + 1% AgVO_3_ Day 1: 9.5 ± 0.9 ppm Day 7: 20 ± 2 ppm Day 14: 10 ± 1 ppm Day 21: 6 ± 1 ppm Day 28: 4.8 ± 0.3 ppm Riva Self Cure + 2.5% AgVO_3_ Day 1: 9.7 ± 0.7 ppm Day 7: 20 ± 2 ppm Day 14: 10.4 ± 0.8 ppm Day 21: 6.2 ± 0.8 ppm Day 28: 4.9 ± 0.4 ppm Riva Self Cure + 5% AgVO_3_ Day 1: 9.2 ± 0.7 ppm Day 7: 19 ± 2 ppm Day 14: 12 ± 2 ppm Day 21: 5.2 ± 0.7 ppm Day 28: 4.9 ± 0.7 ppm	Ag^+^ and V^4+^/V^5+^	N/A
Guo [55]	Ketac Molar Easymix (conventional GIC)	AgNW and AgNP silver nanowire and silver nanoparticles	GIC (0), AgNW-GIC (0.05, 0.1, 0.3, 0.5), AgNP-GIC (0.5) wt%	Ion chromatograph (IC)	deionized water	No numeric data	N/A	The compressive strength ± SD: - GIC (45.83 ± 3.13) MPa - 0.05% AgNW-GIC (40.00 ± 5.76) MPa - 0.1% AgNW-GIC (40.00 ± 4.05) MPa - 0.3% AgNW-GIC (42.67 ± 2.34) MPa - 0.5% AgNW-GIC (41.67 ± 1.51) MPa - 0.5% AgNP-GIC (44.33 ± 2.42) MPa Microhardness ± SD (HV): - GIC (541.2 ± 5.8) kg/mm^2^ - 0.05% AgNW-GIC (529.5 ± 6.6) kg/mm^2^ - 0.1% AgNW-GIC (492.6 ± 9.7) kg/mm^2^ - 0.3% AgNW-GIC (487.0 ± 1.2) kg/mm^2^ - 0.5% AgNW-GIC (470.9 ± 5.3) kg/mm^2^ - 0.5% AgNP-GIC (457.8 ± 2.7) kg/mm^2^
Raghimi [56]	Fuji II LC (RMGIC)	Ag/HA/Si silver hydroxyapatite-silica hybrid na noparticles	0, 0.1, 0.5, 1 and 2 wt%	FISE	distilled water	0% GI (mean ± SD) Day 1: 0.11 ± 0.35 ppm Day 2: 0.11 ± 0.35 ppm Day 3: 0.86 ± 0.35 ppm Day 7: 0.11 ± 0.10 ppm Day 14: 0.08 ± 0.10 ppm Day 28: 0.05 ± 0.15 ppm 0.1% GI (mean ± SD) Day 1: 0.08 ± 0.25 ppm Day 2: 0.10 ± 0.22 ppm Day 3: 0.11 ± 0.15 ppm Day 7: 0.11 ± 0.10 ppm Day 14: 0.10 ± 0.10 ppm Day 28: 0.08 ± 0.30 ppm 0.5% GI (mean ± SD) Day 1: 0.10 ± 0.02 ppm Day 2: 0.12 ± 0.01 ppm Day 3: 0.12 ± 0.00 ppm Day 7: 0.15 ± 0.10 ppm Day 14: 0.17 ± 0.10 ppm Day 28: 0.17 ± 0.57 ppm 1% GI (mean ± SD) Day 1: 0.15 ± 0.06 ppm Day 2: 0.13 ± 0.04 ppm Day 3: 0.18 ± 0.01 ppm Day 7: 0.23 ± 0.03 ppm Day 14: 0.27 ± 0.01 ppm Day 28: 0.32 ± 0.02 ppm 2% GI (mean ± SD) Day 1: 0.22 ± 0.02 ppm Day 2: 0.34 ± 0.04 ppm Day 3: 0.35 ± 0.05 ppm Day 7: 0.52 ± 0.02 ppm Day 14: 0.64 ± 0.09 ppm Day 28: 0.63 ± 0.45 ppm	N/A	The compressive strength (mean ± SD): - 0 wt% (97.14 ± 31.56) MPa - 0.1 wt% (97.84 ± 25.73) MPa - 0.5 wt% (100.65 ± 42.29) MPa - 1 wt% (109.5 ± 34.70) MPa - 2 wt% (89.33 ± 21.57) MPa
Qasim [57]	Riva Selfcure (conventional GIC)	MSAgNP mesoporous silica with silver nanoparticles	0, 1, 3 and 5 wt%	High-Performance Liquid Chromatography (HPLC)	distilled water	No numeric data	Al^3+^, Ca ^2+^, Na^+^, P^3−^, Ag^+^	Glass ionomer cements modified with mesoporous silica and silver nanoparticles exhibited microhardness similar to conventional GICs.
AlMatar [59]	RMGI (Fuji PLUS)	AgNP ZnONP AgNP+ZnONP	5 wt	High-Performance Liquid Chromatography (HPLC)	distilled water	At day 2: - control = 3.84 ± 0.88 mg/L - 5% AgNP = 5.15 ± 0.36 mg/L - 5%AgNP+5%ZnONP = 3.87 ± 0.04 mg/L	N/A	After 14 days: Vickers microhardness: - control (27.22 ± 0.99) g/μm^2^ - 5% ZnONP (24.89 ± 1.33) g/μm^2^ After 14 days 5% ZnONP comparable to control. Other experimental samples had lower microhardness values to control.
Gunay [61]	- metal reinforced GI (Riva Silver) - metal reinforced GI (ChemFil Rock)	silver-alloy calcium-aluminium-zinc-fluoro-phosphorus-silicate glass	No data	FISE	distilled water	Riva Silver: Day 1 = 3.21 ± 1.96 mg/L Day 2 = 5.47 ± 1.75 mg/L Day 3 = 5.81 ± 1.99 mg/L Day 7 = 9.66 ± 2.53 mg/L Day 14 = 13.34 ± 3.08 mg/L Day 21 = 16.44 ± 4.09 mg/L Day 28 = 18.00 ± 4.09 mg/L ChemFil Rock: Day 1 = 2.16 ± 0.97 mg/L Day 2 = 2.64 ± 1.36 mg/L Day 3 = 2.16 ± 0.77 mg/L Day 7 = 4.14 ± 0.97 mg/L Day 14 = 4.99 ± 1.58 mg/L Day 21 = 6.35 ± 1.82 mg/L Day 28 = 6.73 ± 1.77 mg/L	N/A	N/A
Wassel [62]	Conventional self-cure GIC (Riva, SDI)	AgNP TiO_2_NP	5 wt	FISE	deionized water	Cumulative values after 28 days: Control—0.056 ± 078 mg/cm^2^ AgNP—0.065 ± 0.157 mg/cm^2^ TiO_2_NP—0.0470 ± 0.056 mg/cm^2^	N/A	Compressive strength: Control: 136.48 ± 13.40 MPa Ag: 144.32 ± 14.95 MPa Ti: 166.31 ± 15.08 MPa Compressive strength values are higher when metals are added to the material.
Alshehri [63]	conventional GIC (GC Fuji II)	AgNP	0.1 and 0.2	FISE	deionized water.	No numeric data	N/A	N/A
Selimovic-Dragas [72]	GIC (Ketac Silver)	Ag	No data	FISE	Distilled water	After 8 h = 0.150 (0.106) ug/g After 24 h = 0.229 (0.133) ug/g	N/A	N/A
Xu [73]	Ketac Silver, Miracle Mix,	sintered Ag, AgSnCu alloy	N/A	FISE	3 mL of deionized water	Ketac Silver: 318 ± 47 µg/cm^2^ Miracle Mix 398 ± 32 µg/cm^2^	N/A	Comprehensive strength- resulted in lower properties than other GIC, compomers or composites.
Helvatjoglu-Antoniades [75]	- GIC: Fuji III (FIII), Ketac-Cem (KC) - Metal-reinforced GIC: Miracle Mix (MM), Ketac-Silver (KS) - RMGIC: Fuji II LC (FII LC) - Compomer: Compoglass Flow (COM) - Sealants: Fissurit F (FS), Helioseal F (HL) - Composite resin: Tetric (TE)	Miracle Mix (MM) additive: silver alloy Ketac-Silver (K) additive: sintered silver (Ag)	MM: Silver (>50% m/m), tin (<30% m/m), copper (>10% m/m), the powder consists of calcium alumino fluoro silicate glass (<50% m/m) mixed with silver alloy K: Calcium alumino fluoro silicate glass mixed with sintered silver in a ratio of 0.92:1; 48% silver content in the powder	FISE	7 mL of double distilled water, 37 °C per sample	Total amount of fluoride (cumulative over 112 days ranked from highest to lowest): MM: 11.7 µg/mm^2^, F III (Fuji III): 8.3 µg/mm^2^, KC: 7.1 µg/mm^2^, F II LC: 4.7 µg/mm^2^ KS: 3.1 µg/mm^2^ COM: 2.6 µg/mm^2^ FS: 0.9 µg/mm^2^ HL: 0.6 µg/mm^2^ TE: 0.1 µg/mm^2^	not tested	not tested
Hattab [76]	GIC: Ketac-Fil (KF) and Fuji II (FJ) Metal-reinforced GIC: Ketac-Silver (KS)	KS: sintered silver (Ag)	KS: 40% of the fluoride-containing glass is replaced by silver with ratio of 0.92:1	FISE	50 mL, 37 °C of each solution: deionized water, artificial saliva (pH 5.5), aqueous solution of hydroxyapatite	Cumulative release over 28 days: In deionized water: - FJ: 405 μg/cm^2^ - KF: 391 μg/cm^2^ - KS: 132 μg/cm^2^ In artificial saliva: FJ: 148 μg/cm^2^ (36.5% of release in deionized water) KF: 161 μg/cm^2^ (41.1% of release in deionized water) KS: 49 μg/cm^2^ (36.9% of release in deionized water) Fluoride uptake by hydroxyapatite from GIC samples after 14 days: - FJ + varnish: 2167 ± 56 μg/g - FJ + resin coating: 686 ± 37 μg/g - KF + varnish: 506 ± 30 μg/g - KF + resin coating: 387 ± 17 μg/g - KS + varnish: 166 ± 11 μg/g - KS + resin coating: 368 ± 24 μg/g	not tested	not tested
Karimi [68]	RMGIC (SFAG-ACP powder and GC Fuji LININGTM LC liquid)	melt-derived strontium fluoro-aluminosilicate glass (SFAG)	0, 1.5, 3, 5, 10 and 20%	FISE	Distilled water	0% Day 1 = 98.55 Day 7 = 28.76 Day 14 = 19.39 Day 28 = 16.37 1.5% Day 1 = 96.58 Day 7 = 29.77 Day 14 = 19.33 Day 28 = 16.53 3% Day 1 = 91.04 Day 7 = 28.74 Day 14 = 19.01 Day 28 = 16.33 5% Day 1 = 80.59 Day 7 = 22.76 Day 14 = 13.37 Day 28 = 12.96 10% Day 1 = 61.02 Day 7 = 16.67 Day 14 = 11.75 Day 28 = 9.78 20% Day 1 = 33.87 Day 7 = 13.07 Day 14 = 9.76 Day 28 = 7.69	Ca2+, PO43−	Comprehensive strength: 0% Day 0 = 111.05 Day 1 = 113.12 Day 3 = 117.22 Day 7 = 116.04 Day 14 = 223.02 Day 28 = 300.68 1.5% Day 0 = 115.22 Day 1 = 113.06 Day 3 = 118.08 Day 7 = 118.14 Day 14 = 200.88 Day 28 = 280.98 3% Day 0 = 113.35 Day 1 = 116.66 Day 3 = 116.27 Day 7 = 117.32 Day 14 = 200.17 Day 28 = 280.42 5% Day 0 = 114.44 Day 1 = 110.83 Day 3 = 115.43 Day 7 = 116.19 Day 14 = 188.49 Day 28 = 272.23 10% Day 0 = 107.09 Day 1 = 101.98 Day 3 = 105.35 Day 7 = 110.64 Day 14 = 145.36 Day 28 = 204.25 20% Day 0 = 103.39 Day 1 = 104.77 Day 3 = 105.29 Day 7 = 107.33 Day 14 = 134.07 Day 28 = 192.24
Thongsri [60]	Synthesized sol-gel glass ionomer (SGIC)	bioactive glass powder with SrF2	0, 1, 3, 6 wt	FISE	distilled water	No numeric data	Ca, Al, Si	No numeric data. Compressive strength higher with 1% BGF added than SGIC without BGF.
Potiprapanpong [58]	Experimental RMGIC	HEMA (2-hydroxyethyl methacrylate) and Sr/F-BGNPs (bioactive glass nanoparticles)	HEMA: 0 or 5 wt%, Sr/F-BGNPs: 5 or 10 wt%	FISE (Orion)	deionized water	The highest cumulative fluoride release (at 4 weeks) was observed for H5S10 (137.5 ± 6.1 ppm). The cumulative fluoride release values for the other materials were as follows: H5S5 (136.6 ± 2.2 ppm), H0S10 (72.6 ± 3.0 ppm) and H0S5 (73.0 ± 9.4 ppm).	Al, Ca, P, and Sr	Biaxial Flexural Strength (BFS ± SD): - H5S10 (11 ± 1) MPa - H5S5 (31 ± 4) MPa - H0S10 (16 ± 2) MPa - H0S5 (24 ± 3) MPa Modulus (BFM ± SD): - H5S10 (0.03 ± 0.02) GPa - H5S5 (0.93 ± 0.29) GPa - H0S10 (0.15 ± 0.09) GPa - H0S5 (0.79 ± 0.09) GPa
Shahid [71]	GIC	SrO and SrF2	G1 = 0% SrF2, 0% SrO G2 = 2% SrF2, 3% SrO G3 = 2% SrF2, 1.5% SrO G4 = 2% SrF2, 0.5% SrO G5 = 1% SrF2, 0% SrO G6 = 1% SrF2, 1.5% SrO G7 = 1.5% SrF2, 1% SrO G8 = 0.5% SrF2, 2% SrO G9 = 0.25% SrF2, 2.25% SrO G10 = 0% SrF2, 2.5% SrO	FISE	Acetic acid at pH 4	G1 = 0.56 mequiv/g G2 = 0.93 mequiv/g G3 = 1.11 mequiv/g G4 = 0.75 mequiv/g G5 = 0.47 mequiv/g G6 = 0.8 mequiv/g G7 = 2.61 mequiv/g G8 = 0.29 mequiv/g G9 = 0.21 mequiv/g	Sr^2+^, Ca^2+^ and Al^3+^	N/A
Saxena [70]	GIC (Zirconomer)	ZrO2	No data	FISE	Artificial saliva	Day 1 = 29.38 ppm Day 3 = 31.69 ppm Day 7 = 35.65 ppm Day 15 = 25.58 ppm Day 30 = 9.46 Control (Fuji IX) Day 1 = 13.72 ppm Day 3 = 15.08 ppm Day 7 = 15.46 ppm Day 15 = 7.39 ppm Day 30 = 2.53 ppm	N/A	N/A
Cibim [69]	GIC + TiO2 nanotubes	TiO2	0, 3, 5 and 7%	FISE	Demineralizing and remineralizing solutions	DE solution Control Day 1 = 0.198 (0.05) ppm Day 2 = 0.156 (0.04) ppm Day 3 = 0.145 (0.05) ppm Day 5 = 0.141 (0.05) ppm Day 7 = 0.141 (0.05) ppm Day 9 = 0.124 (0.04) ppm Day 12 = 0.159 (0.04) ppm Day 15 = 0.165 (0.07) ppm 3% Day 1 = 0.298 (0.07) ppm Day 2 = 0.233 (0.06) ppm Day 3 = 0.199 (0.03) ppm Day 5 = 0.179 (0.02) ppm Day 7 = 0.179 (0.03) ppm Day 9 = 0.158 (0.03) ppm Day 12 = 0.168 (0.04) ppm Day 15 = 1.172 (0.04) ppm 5% Day 1 = 0.292 (0.08) ppm Day 2 = 0.256 (0.08) ppm Day 3 = 0.197 (0.06) ppm Day 5 = 0.187 (0.05) ppm Day 7 = 0.212 (0.06) ppm Day 9 = 0.155 (0.05) ppm Day 12 = 0.213 (0.04) ppm Day 15 = 0.213 (0.04) ppm 7% Day 1 = 0.311 (0.08) ppm Day 2 = 0.249 (0.09) ppm Day 3 = 0.191 (0.05) ppm Day 5 = 0.181 (0.05) ppm Day 7 = 0.168 (0.05) ppm Day 9 = 0.154 (0.04) ppm Day 12 = 0.171 (0.04) ppm Day 15 = 0.164 (0.06) ppm RE solution Control Day 1 = 0.049 (0.01) ppm Day 2 = 0.03 (0.01) ppm Day 3 = 0.033 (0.01) ppm Day 5 = 0.027 (0.01) ppm Day 7 = 0.03 (0.01) ppm Day 9 = 0.032 (0.01) ppm Day 12 = 0.031 (0.01) ppm Day 15 = 0.031 (0.01) ppm 3% Day 1 = 0.041 (0.01) ppm Day 2 = 0.029 (0.01) ppm Day 3 = 0.037 (0.01) ppm Day 5 = 0.036 (0.01) ppm Day 7 = 0.033 (0.01) ppm Day 9 = 0.037 (0.01) ppm Day 12 = 0.034 (0.01) ppm Day 15 = 0.039 (0.01) ppm 5% Day 1 = 0.037 (0.01) ppm Day 2 = 0.035 (0.02) ppm Day 3 = 0.036 (0.03) ppm Day 5 = 0.04 (0.01) ppm Day 7 = 0.038 (0.01) ppm Day 9 = 0.042 (0.01) ppm Day 12 = 0.037 (0.01) ppm Day 15 = 0.047 (0.01) ppm 7% Day 1 = 0.067 (0.02) ppm Day 2 = 0.047 (0.02) ppm Day 3 = 0.043ppm Day 5 = 0.041 (0.01) ppm Day 7 = 0.039 (0.01) ppm Day 9 = 0.044 (0.01) ppm Day 12 = 0.045 (0.02) ppm Day 15 = 0.044 (0.02) ppm	N/A	Surface roughness Control = 0.41 ± 0.14 3% = 0.55 ± 0.17 5% = 0.49 ± 0.07 7% = 0.58 ± 0.16 Surface hardness Control = 81.48 ± 9.87 3% = 105.87 ± 12.71 5% = 118.25 ± 4.21 7% = 75.13 ± 6.61
Putri [67]	GIC with ZnO nanoparticles	ZnO	10 or 15%	Spectrophotometer	Distilled water	Control = 0.417 (0.133) ppm 10% = 0.571 (0.099) ppm 15% = 0.457 (0.144) ppm	N/A	N/A
Bahammam [66]	GIC (ChemFil ROCK)	calcium-aluminum-zinc-fluoro-phosphor-silicate glass	No data	FISE (Fisher Scientific Accumet 13-620–629)	Distilled water	1.68 ± 0.08 μg/cm^2^	O, F, Na, Mg, Al, Si, P, S, Ca, Sr, Zn, and Zr	N/A
Malekhoseini [65]	RMGI (Fuji II LC + ZnO nanoparticles)	ZnO	0%, 1%, 2%, 3% or 4%	FISE and potentiometer	Deionized water	3% = 34.6 ppm 0% = 35 ppm	Zn	Flexural strength: no numeric data Flexural modulus: no numeric data Micro shear bond strength: Control: Day 1 = 10.8 ± 2.2 MPa Day 7 = 12.36 ± 3.6 MPa Day 30 = 16.76 ± 5.82 MPa 2%: Day 1 = 10.96 ± 3.72 MPa Day 7 = 14.63 ± 2.56 MPa Day 30 = 12.1 ± 2.78 MPa
Kohno [64]	GIC with BioUnion Filler	Fluorozincsilicate glass	No data	FISE	Artificial saliva + acetate buffer solution (pH 4.5)	No numeric data	Zn	N/A
Osinaga [74]	GIC: Ketac-Fil RMGIC: Vitremer	ZnSO_4_	5% ZnSO_4_, 10% ZnSO_4_	FISE	Artificial saliva (1 mL), 100% humid environment, 37 °C	For Ketac-Fil groups: - K0 (Ketac-Fil control, no ZnSO_4_): from initial: 35 ppm to 5 ppm by day 5 - K5 (Ketac-Fil with 5% ZnSO_4_): from initial: 25 ppm to 7 ppm by day 5 - K10 (Ketac-Fil with 10% ZnSO_4_): from initial 15 ppm, to 9 ppm by day 5 For Vitremer groups: - V0 (Vitremer control, no ZnSO_4_): from initial 32 ppm to 10 ppm by day 5 - V5 (Vitremer with 5% ZnSO_4_): from initial 23 ppm to 6 ppm by day 5 - V10 (Vitremer with 10% ZnSO_4_): from initial 16 ppm to 3 ppm by day 5 For recharged with F (after day 15): - K0-F: from 5–6 ppm at day 15 to 1 ppm by day 30 - K5-F: from 4–5 ppm at day 15 to 1 ppm by day 30 - K10F: from 4–5 ppm at day 15 to 1 ppm by day 30 - V0-F: from 4–5 ppm at day 15 to 1 ppm by day 30 - V5-F: from 3–4 ppm at day 15 to 1 ppm by day 30 - V10-F: from 3–4 ppm at day 15, to 1 ppm by day 30 - For recharged with F + Zn (after day 15): - K0-F + Zn: from 10 ppm at day 15 to 1 ppm by day 30 - K5-F + Zn: from 6 ppm at day 15 to 1 ppm by day 30 - K10-F + Zn: from 5 ppm at day 15 to 1 ppm by day 30 - V0-F + Zn: from 3 ppm at day 15 to 1 ppm by day 30 - V5-F + Zn: from 2–3 ppm at day 15 to 1 ppm by day 30 - V10-F + Zn: from 2 ppm at day 15 to 1 ppm by day 30	Zinc (Zn)—measured by inductively coupled argon plasma emission spectrometry	Flexural strength: - Ketac-Fil control: 30.7 MPa - Ketac-Fil with 5% ZnSO_4_: 29.7 MPa - Ketac-Fil with 10% ZnSO_4_: 27.7 MPa - Vitremer control: 65.0 MPa - Vitremer with 5% ZnSO_4_:62.7 MPa - Vitremer with 10% ZnSO_4_: 64.2 MPa

**Table 3:** Quality assessment—JBI checklist for quasi-experimental studies (nonrandomized experimental studies).

Authors	(1) Is It Clear in the Study What Is the ‘Cause’ and What Is the ‘Effect’?	(2) Were the Participants Included in Any Comparisons Similar?	(3) Were the Participants Included in Any Comparisons Receiving Similar Treatment/Care, Other than the Exposure or Intervention of Interest?	(4) Was There a Control Group?	(5) Were There Multiple Measurements of the Outcome Both Pre and Post the Intervention/Exposure?	(6) Was Follow up Complete and If Not, Were Differences Between Groups in Terms of Their Follow Up Adequately Described and Analyzed?	(7) Were the Outcomes of Participants Included in Any Comparisons Measured in the Same Way?	(8) Were Outcomes Measured in a Reliable Way?	(9) Was Appropriate Statistical Analysis Used?
Pardi [54]	Yes	Yes	Yes	Yes	Yes	Yes	Yes	Yes	Yes
Guo [55]	Yes	Yes	Yes	Yes	No	Yes	Yes	Yes	Yes
Raghimi [56]	Yes	Yes	Yes	Yes	No	Yes	Yes	Yes	Yes
Qasim [57]	Yes	Yes	Yes	Yes	No	Yes	Yes	Yes	Yes
Potiprapanpong [58]	Yes	Yes	Yes	Yes	No	Yes	Yes	Yes	Yes
AlMatar [59]	Yes	Yes	Yes	Yes	No	Yes	Yes	Yes	Yes
Thongsri [60]	Yes	Yes	Yes	Yes	No	Yes	Yes	Yes	Yes
Gunay [61]	Yes	Yes	Yes	Yes	No	Yes	Yes	Yes	Yes
Wassel [62]	Yes	Yes	Yes	Yes	No	Yes	Yes	Yes	Yes
Alshehri [63]	Yes	Yes	Yes	Yes	No	Yes	Yes	Yes	Yes
Kohno [64]	Yes	Yes	Yes	Yes	No	Yes	Yes	Yes	Yes
Malekhoseini [65]	Yes	Yes	Yes	Yes	No	Yes	Yes	Yes	Yes
Bahammam [66]	Yes	Yes	Yes	Yes	No	Yes	Yes	Yes	Yes
Putri [67]	Yes	Yes	Yes	Yes	No	Yes	Yes	Yes	Yes
Karimi [68]	Yes	Yes	Yes	Yes	No	Yes	Yes	Yes	Yes
Cibim [69]	Yes	Yes	Yes	Yes	No	Yes	Yes	Yes	Yes
Saxena [70]	Yes	Yes	Yes	Yes	Yes	Yes	Yes	Yes	Yes
Shahid [71]	Yes	Yes	Yes	Yes	No	Yes	Yes	Yes	Yes
Selimovic-Dragas [72]	Yes	Yes	Yes	Yes	No	Yes	Yes	Yes	Yes
Xu [73]	Yes	Yes	Yes	Yes	No	Yes	Yes	Yes	Yes
Osinaga [74]	Yes	Yes	Yes	Yes	No	Yes	Yes	Yes	Yes
Helvatjoglu-Antoniades [75]	Yes	Yes	Yes	Yes	No	Yes	Yes	Yes	Yes
Hattab [76]	Yes	Yes	Yes	Yes	No	Yes	Yes	Yes	Yes

## Data Availability

No new data were created or analyzed in this study. Data sharing is not applicable to this article.

## Supplementary Materials

The following supporting information can be downloaded at: https://www.mdpi.com/article/10.3390/ma18133187/s1, Table S1: PRISMA 2020 Checklist.
